# Nine-Year Follow-Up of GAD65 Antibody Limbic Encephalitis With Clinical Remission Despite Persistent Imaging and Serological Abnormalities

**DOI:** 10.7759/cureus.85726

**Published:** 2025-06-10

**Authors:** Thanda Aung, Benjamin E Plotkin

**Affiliations:** 1 Rheumatology, David Geffen School of Medicine at University of California Los Angeles, Los Angeles, USA; 2 Radiology, David Geffen School of Medicine at University of California Los Angeles, Los Angeles, USA

**Keywords:** autoimmune encephalitis, autoimmune limbic encephalitis, gad65 antibody, hydroxychloroquine, mycophenolate, mycophenolate mofetil (mmf), rituximab

## Abstract

Glutamic acid decarboxylase 65 (GAD65) antibody-associated autoimmune encephalitis is a rare neurological disorder characterized by cognitive impairment, seizures, and psychiatric manifestations. Long-term outcomes and management strategies for this condition remain poorly documented in the medical literature. We present a nine-year follow-up of a young woman with GAD65 antibody-positive autoimmune limbic encephalitis who achieved complete clinical remission following aggressive immunomodulatory therapy. The patient, initially treated with rituximab, intravenous immunoglobulin (IVIG), and mycophenolate, successfully discontinued all immunosuppressive medications except hydroxychloroquine. She remains clinically asymptomatic with no recurrence of olfactory hallucinations or other neurological deficits, despite persistently elevated serum GAD65 antibody levels and persistent signal abnormalities on brain imaging. This case highlights the potential for favorable long-term outcomes in GAD65 antibody-associated encephalitis with appropriate immunotherapy and provides insights into maintenance therapy and medication withdrawal strategies for patients planning pregnancy.

## Introduction

Glutamic acid decarboxylase 65 (GAD65) autoimmune limbic encephalitis is a subtype of autoimmune encephalitis characterized by the presence of antibodies against the 65 kDa isoform of glutamic acid decarboxylase (GAD). This enzyme is crucial for the synthesis of gamma-aminobutyric acid (GABA), the primary inhibitory neurotransmitter in the central nervous system [1].

Patients with GAD65 autoimmune limbic encephalitis typically present with a range of neuropsychiatric symptoms, including anterograde amnesia, temporal lobe seizures, and various psychiatric manifestations such as anxiety, depression, and behavioral changes [1]. The condition predominantly affects young to middle-aged women and often has a subacute or chronic course [2].

Treatment primarily involves immunotherapy, including corticosteroids, intravenous immunoglobulin (IVIG), and plasma exchange. These interventions aim to reduce antibody levels and alleviate symptoms. However, the response to immunotherapy can be variable, with some patients requiring prolonged treatment [2]. We present a nine-year follow-up of a previously reported case of GAD65 antibody-positive limbic encephalitis [3], highlighting the disease course, therapeutic decisions, clinical-serological dissociation, and specific considerations for medication management during family planning.

## Case presentation

A 35-year-old Caucasian female with idiopathic pulmonary arterial hypertension initially presented in 2016 with a two-week history of olfactory hallucinations described as "chemical smell," short-term memory deficits, emotional lability, disorientation, and intermittent left-sided facial and body numbness. She had no features suggesting lupus or other autoimmune rheumatological diseases, no family history of autoimmune disease, and no recent travel or illness. On examination, there were no neurological deficits, and the rest of the systemic examination was unremarkable.

Laboratory investigations showed elevated inflammatory markers: erythrocyte sedimentation rate 72 mm/hour (reference range <25 mm/hr) and C-reactive protein 1.6 mg/dL (reference range <0.3 mg/dL); however, complete blood count and comprehensive metabolic profile were normal. Serological testing revealed positive antinuclear antibodies (ANA) with nucleolar pattern at 1:320 titer. Extensive workup for lupus and other autoimmune connective tissue diseases was unremarkable (Table 1). Additional serologies, including anti-N-methyl-D-aspartate (NMDA) receptor antibody, anti-voltage-gated potassium channel (VGKC) complex antibody, anti-Hu, anti-Yo, anti-Ri, and other paraneoplastic panels, were all normal (Table 1).

**Table 1:** Key laboratory values GAD65: glutamic acid decarboxylase 65; NMDA: N-methyl-D-aspartate; VGKC: voltage-gated potassium channels, Paraneoplastic neurology panel: Anti-Hu (ANNA-1), Anti-Yo (PCA-1), Anti-Ri (ANNA-2), Anti-Ma2, Anti-CV2 (CRMP5), Anti-amphiphysin, Anti-Tr (DNER), Anti-GAD65, and Anti-NMDA receptor antibodies.

Parameters	Patient Values	Reference Ranges
Serum GAD65 antibody	>250 U/mL	<5.0 U/mL
Antinuclear antibody (ANA)	1:320 (nucleolar pattern)	<1:40
Complete blood count	Within normal limits	--
Comprehensive metabolic panel	Within normal limits	--
Erythrocyte sedimentation rate	72 mm/hr	<25 mm/hr
C-reactive protein	1.6 mg/dL	<0.3 mg/dL
Thyroid function tests	Within normal limits	--
Other paraneoplastic antibody panel	Negative	Negative
Serum protein electrophoresis	Normal pattern	--
Anti-NMDA receptor antibody	Negative	Negative
Anti-VGKC complex antibody	Negative	Negative
Anti-Hu, Anti-Yo, Anti-Ri antibodies	Negative	Negative

**Figure 1:**
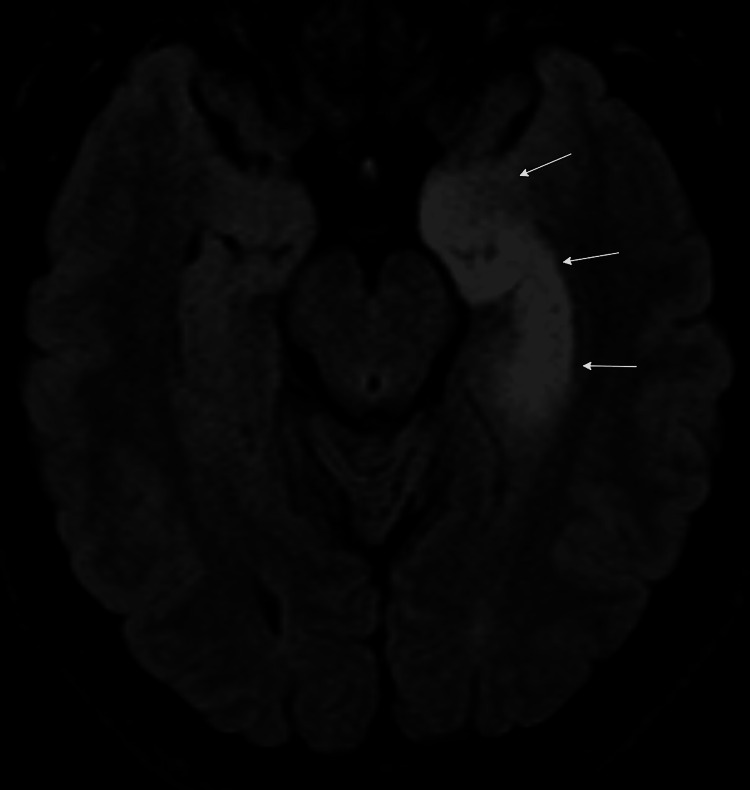
Brain magnetic resonance imaging, axial FLAIR sequences showing increase signal within the left mesiotemporal lobe (arrows), 2016 FLAIR: fluid attenuated inversion recovery

Electroencephalography (EEG) revealed left mesial temporal seizures, while brain MRI demonstrated edema and abnormal enhancement in the left temporal lobe, predominantly affecting the hippocampal formation (Figure 1). Cerebrospinal fluid analysis showed high-titer GAD65 antibodies, with correspondingly elevated serum GAD65 antibodies (Table 2).

**Table 2:** Cerebrospinal fluid analysis IgG: immunoglobulin G; GAD65: glutamic acid decarboxylase 65; PCR: polymerase chain reaction

Parameters	Patient Values	Reference Ranges
Opening pressure	18 cm H₂O	10-20 cm H₂O
Appearance	Clear, colorless	Clear, colorless
WBC count	8 cells/μL	0-5 cells/μL
RBC count	0 cells/μL	0 cells/μL
Protein	42 mg/dL	15-45 mg/dL
Glucose	65 mg/dL	45-80 mg/dL
HSV Type 1 DNA	Not Detected	Negative
HSV Type 2 DNA	Not Detected	Negative
Oligoclonal bands	Present	Absent
IgG index	0.8	<0.7
GAD65 antibody	1:100	Negative
Cytology	Negative for malignant cells	Negative
Viral PCR panel	Negative	Negative
Bacterial culture	No growth	No growth

The patient underwent a full-body PET scan as well as CT imaging of the chest, abdomen, and pelvis to identify any underlying neoplasm causing a paraneoplastic syndrome associated with GAD65 antibodies; however, no malignancy was identified. The infectious disease workup was unremarkable. The patient was diagnosed with GAD65 antibody-positive autoimmune limbic encephalitis.

Initial treatment included a three-day course of high-dose pulse methylprednisolone (1 gram daily), subsequently a four-day course of IVIG, followed by oral prednisone (60 mg daily, 1 mg/kg body weight, with taper). The patient demonstrated excellent clinical response within one week, with substantial reduction in olfactory hallucinations and significant improvement in cognitive function and memory. She was subsequently maintained on rituximab (1000 mg, two doses, two weeks apart, administered every six months), monthly IVIG (0.5 gram/kg body weight weekly), and oral mycophenolate (1500 mg twice daily). For seizure management, she was initially prescribed multiple anti-epileptic medications, including levetiracetam 2g twice daily, lacosamide 200 mg twice daily, and zonisamide 150 mg at bedtime. By three months post-treatment initiation, she had returned to work with complete resolution of disorientation, memory deficits, and numbness, though she continued to experience infrequent episodes of transient olfactory hallucinations over the initial five years with gradual continuing improvement.

**Figure 2:**
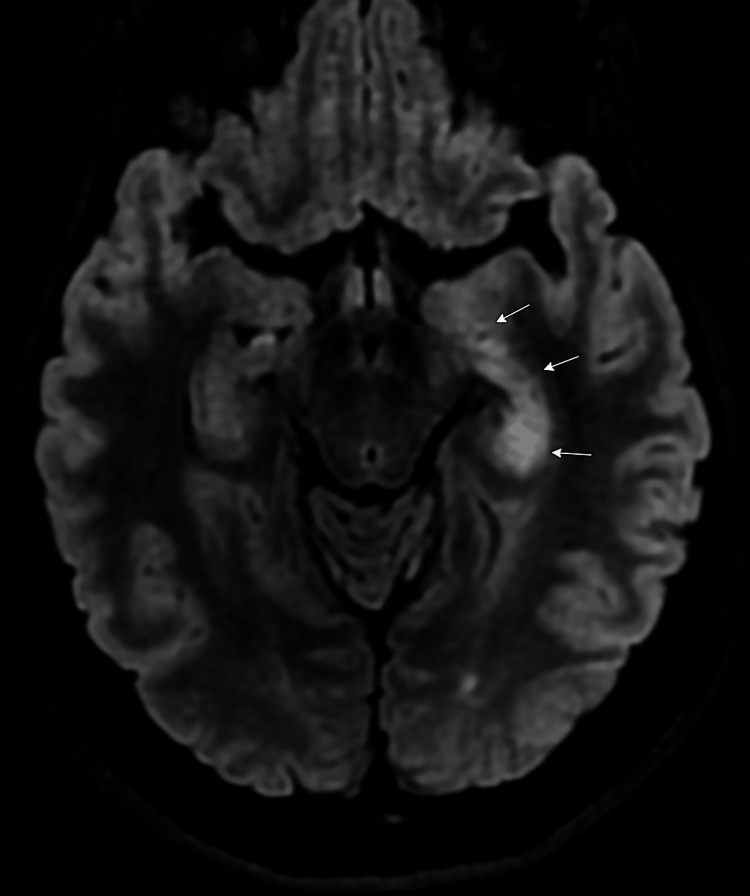
Brain magnetic resonance imaging, axial FLAIR sequences showing increase signal within the left mesiotemporal lobe (arrows), 2018 FLAIR: fluid attenuated inversion recovery

**Figure 3:**
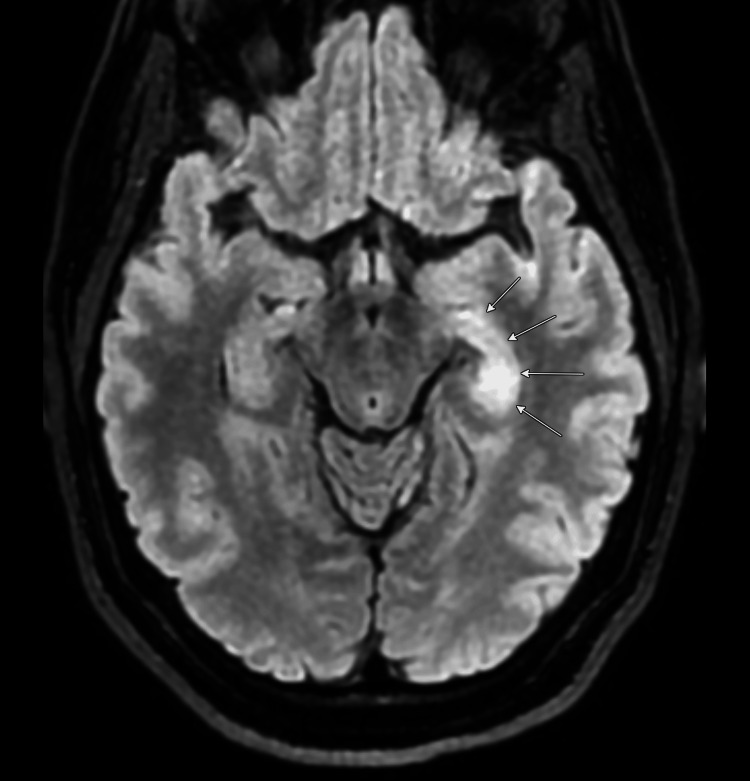
Brain magnetic resonance imaging, axial FLAIR sequences showing increase signal within the left mesiotemporal lobe (arrows), 2024 FLAIR: fluid attenuated inversion recovery

Now, nine years later, in 2025, the patient demonstrates no further olfactory hallucinations, no memory deficits, and has successfully undergone gradual tapering of immunosuppressive therapy. IVIG was discontinued after eight years, and rituximab was continued for a total of eight years, with the last infusion administered in 2024. Her anti-epileptic regimen has been simplified to levetiracetam monotherapy. Mycophenolate was tapered and ultimately discontinued after six years, specifically due to the patient's desire to conceive and her current in vitro fertilization process. Mycophenolate is contraindicated during pregnancy due to teratogenicity, and she was transitioned to hydroxychloroquine 200 mg daily in year seven as maintenance therapy due to its established safety profile during pregnancy. Current laboratory studies show normal inflammatory markers, although persistently high serum GAD65 antibody levels remain (>250 U/mL). Serial brain MRIs have shown diminished size of signal intensity but persistent abnormalities within the left mesiotemporal lobe (Figures 2, 3). Most notably, the patient remains clinically asymptomatic on hydroxychloroquine monotherapy despite the persistence of elevated GAD65 antibody levels and persistent abnormal signal within the left temporal lobe, highlighting the dissociation between serological markers and clinical manifestations in this condition.

## Discussion

GAD65 encephalitis is a form of autoimmune encephalitis characterized by the presence of antibodies against the GAD65 enzyme. This condition can present with a variety of neuropsychiatric symptoms, including seizures, memory deficits, and psychiatric manifestations such as anxiety and depression. A systematic review by Vrillon et al. identified 21 cases of anti-GAD65-associated limbic encephalitis with neuropsychiatric signs. The median age at onset was 27 years, with a female predominance (81%). Common clinical features included anterograde amnesia and seizures, with psychiatric symptoms present in 61.9% of patients [1]. Kuang et al. reported on meningoencephalitis associated with GAD65 autoimmunity, identifying three male patients with acute or subacute onset of confusion, psychosis, cognitive symptoms, and seizures [4]. All patients showed significant improvement following immunotherapy with corticosteroids or intravenous immunoglobulin, like our case.

The prevalence of GAD65 antibody-associated encephalitis is relatively low. According to a population-based study conducted in Olmsted County, Minnesota, the prevalence of GAD65 antibody-associated encephalitis was found to be 1.9 per 100,000 individuals [5]. This study highlighted that GAD65 antibody-associated encephalitis is a rare subtype of autoimmune encephalitis, which itself has a prevalence comparable to that of infectious encephalitis.

GAD65 encephalitis is diagnosed through a combination of clinical evaluation, imaging, and laboratory tests. The key diagnostic criteria include clinical presentation, imaging findings, electroencephalography results, and laboratory tests. Patients typically present with symptoms such as seizures, memory deficits, confusion, and other neuropsychiatric symptoms, which are often indicative of limbic encephalitis [2,4]. Brain MRI often reveals hyperintensities in the temporal lobes and hippocampus on T2-weighted and fluid attenuated inversion recovery (FLAIR) sequences [2]. EEG may show epileptiform discharges, particularly in the temporal lobes [2]. Laboratory tests are crucial for diagnosis, with the presence of high-titer anti-GAD65 antibodies in serum and cerebrospinal fluid (CSF) being essential [2,4]. CSF analysis may show mild pleocytosis and elevated protein levels. Oligoclonal bands may also be present, indicating an inflammatory process [2,4].

Treatment options for GAD65 encephalitis include first-line immunotherapy, plasma exchange, second-line immunotherapy, long-term immunosuppression, and symptomatic treatment. First-line immunotherapy typically involves the use of intravenous corticosteroids (e.g., methylprednisolone) and/or IVIG. Both treatments have shown clinical improvements in patients, although the response rates can vary depending on the clinical phenotype. For instance, intravenous methylprednisolone (IVMP) and IVIG have response rates of 45.56% and 36.71%, respectively, in general GAD65 encephalitis cases [6]. Plasma exchange (PLEX) is another first-line option, particularly useful in severe cases or when there is a poor response to initial immunotherapy. PLEX can help remove pathogenic antibodies from the circulation [7]. For patients who do not respond adequately to first-line treatments, second-line agents such as rituximab and cyclophosphamide are considered. Rituximab, in particular, has shown significant efficacy in refractory cases and may be more effective than mycophenolate mofetil (MMF) [8,9]. Chronic immunosuppressive therapy may be required to maintain remission. Agents such as mycophenolate mofetil (MMF) and azathioprine are commonly used, although their effectiveness can vary [10]. In addition to immunotherapy, symptomatic treatments are essential. This includes antiepileptic drugs for seizure control, muscle relaxants for stiffness, and other supportive measures as needed [11].

In cases of GAD65 encephalitis, it is not uncommon for GAD65 antibody levels to remain elevated even after clinical remission. This phenomenon has been observed in multiple studies and case reports. For instance, a study by Di Giacomo et al. reported a case where a patient with limbic encephalitis had significantly elevated GAD65 antibody titers that decreased but remained high even after clinical improvement and cessation of seizures [8]. Similarly, Madlener et al. found that serum GAD65 antibody levels did not correlate with clinical outcomes in patients with GAD65 antibody-associated neurological syndromes, suggesting that antibody titers may not be a reliable marker for disease activity or remission [12].

The prognosis for a case of GAD65 limbic encephalitis (LE) is generally variable but can be favorable with appropriate treatment. According to a study by Bai et al., most patients with GAD65 antibody-associated neurological disorders, including LE, respond well to immunotherapy [10]. In their cohort, 87% of patients had favorable clinical outcomes, defined as a modified Rankin Score (mRS) of ≤2, and 70.4% experienced clinical improvement with a decline in mRS by at least one point. Several studies have demonstrated that early initiation of immunotherapy in patients with GAD65 limbic encephalitis can lead to favorable clinical outcomes. Bai et al. reported that early immunotherapy led to clinical improvement in a significant proportion of patients with GAD65-associated limbic encephalitis [10].

Our patient's case illustrates several important aspects of GAD65 antibody-associated limbic encephalitis management. First, the excellent response to immunotherapy underscores the importance of prompt treatment initiation. Second, the successful withdrawal of rituximab, IVIG, and mycophenolate without symptom recurrence suggests that some patients may achieve sustained remission following an initial period of intensive immunosuppression. This observation aligns with reports from Bai et al., who reported that early immunotherapy led to clinical improvement in a significant proportion of patients with GAD65-associated limbic encephalitis [10].

The management of autoimmune encephalitis in women of childbearing age presents unique challenges. Mycophenolate is contraindicated during pregnancy due to teratogenicity (FDA category D), necessitating medication changes for patients planning conception [13]. In our patient, the transition from mycophenolate to hydroxychloroquine was made specifically for family planning.

Hydroxychloroquine, an antimalarial with immunomodulatory properties, has a well-established safety profile in pregnancy (FDA category C) and has been extensively studied in pregnant women with systemic lupus erythematosus and other autoimmune conditions [14]. The immunomodulatory effects of hydroxychloroquine include inhibition of toll-like receptors, reduction of proinflammatory cytokine production, inhibition of lysosomal activity, and interference with antigen presentation. These mechanisms collectively suppress autoimmune responses while causing minimal immunosuppression compared to other agents [15].

Several limitations should be acknowledged in this case report. First, as with all case reports, our observations from a single patient cannot be generalized to the broader population of patients with GAD65 antibody-associated encephalitis. Second, while our patient had thorough radiological and serological follow-up, we did not perform serial CSF analyses to monitor potential changes in intrathecal antibody production over time due to the invasive nature of this procedure. Third, the lack of standardized protocols for treatment tapering in GAD65 antibody-associated encephalitis meant that our approach was empirical rather than evidence-based. Finally, the concurrent idiopathic pulmonary arterial hypertension in our patient represents a confounding factor that may have influenced treatment decisions and potentially affected the immune response to therapy. Despite these limitations, the prolonged follow-up period and detailed documentation of clinical, serological, and radiological parameters provide valuable insights into the natural history and treatment outcomes in this rare condition.

## Conclusions

GAD65 antibody-associated limbic encephalitis can achieve sustained clinical remission despite persistent serological and radiological abnormalities, as demonstrated in this nine-year follow-up case. Aggressive initial immunosuppression followed by carefully tapered therapy can maintain disease control with minimal ongoing treatment. The case highlights the disconnect between antibody levels, imaging findings, and clinical status. For women planning pregnancy, transitioning to medications like hydroxychloroquine may maintain remission. Larger prospective studies are needed to establish evidence-based long-term management guidelines for this rare disorder.
